# The Effect of Dental Treatment under General Anesthesia on Quality of Life and Growth and Blood Chemistry Parameters in Uncooperative Pediatric Patients with Compromised Oral Health: A Pilot Study

**DOI:** 10.3390/ijerph17124407

**Published:** 2020-06-19

**Authors:** Gianmaria F. Ferrazzano, Claudia Salerno, Giancarla Sangianantoni, Silvia Caruso, Aniello Ingenito, Tiziana Cantile

**Affiliations:** 1 Department of Neuroscience, Reproductive and Oral Sciences, School of Paediatric Dentistry, University of Naples, Federico II, 80100 Naples, Italy; claudia.salerno@ymail.com (C.S.); giancarla.sangia@yahoo.com (G.S.); ingenito@unina.it (A.I.); tizianacantile@yahoo.it (T.C.); 2 Staff Member of Unesco Chair on Health Education and Sustainable Development, University of Naples, Federico II, 80121 Naples, Italy; 3 Department of Biomedical, Surgical and Dental Sciences, School of Specialization in Pediatric Dentistry, University of Milan, 20142 Milan, Italy; 4 Department of Life, Health and Environmental Sciences, University of L’Aquila, 67100 L’Aquila, Italy; silvia.caruso21@gmail.com

**Keywords:** dental caries, children, oral health-related quality of life, growth, blood chemistry parameters

## Abstract

Background: The effect of untreated dental caries and their treatment under general anesthesia (GA) on the quality of life, growth, and blood chemistry parameters in uncooperative pediatric patients has not been extensively elucidated. The aims are to evaluate the impact of dental treatment under GA on oral health-related quality of life (OHRQoL) in uncooperative pediatric patients with severe dental caries and to assess the effect of dental treatment under GA on children’s weight (Wt), height (Ht), Body Mass Index (BMI), and blood chemistry parameters. Methods: Forty-three uncooperative children aged 3–14 years were selected. OHRQoL, through ECOHIS (Early Childhood Oral Health Impact Scale) and COHRQoL (Child Oral Health-Related Quality of Life) questionnaires, Wt, Ht, BMI, and blood chemistry parameters were measured at baseline and eight months after dental treatment under GA. Results: At follow up, the reductions in the ECHOIS and the COHRQoL components were statistically significant (*p* < 0.0001), there was significant improvement in the anthropometric measures: 76.5% of children increased the percentile curves for weight, 68.6% for height, and 51.4% for BMI; for the blood chemistry parameters: ferritin improved in 68.6% of the samples, PCR in 65.7%, ESR in 68.6%, Vitamin D in 68.6%, and IGF-1 in 65.7%. Conclusions: Oral health status significantly influences OHRQoL, growth, and blood chemistry parameters in uncooperative pediatric patients.

## 1. Introduction

Several studies conducted in hospital centers or educational institutions have noted that caries is a significant problem, especially in early childhood and in children with special health care needs [1,2,3]. Treating an uncooperative child, due to age or systemic illness, who suffers from severe oral diseases, is a challenge for pediatric dentists, especially when extensive and complex treatments are needed. Despite the behavioral techniques to increase compliance and pharmacological sedation techniques, there are cases in which dental rehabilitation under general anesthesia is the only therapeutic choice to provide a safe and effective dental treatment [4]. Under general anesthesia, all required treatments are performed in a single session in hospital, providing efficient services in a safe environment. Furthermore, general anesthesia treatment of an uncooperative child allows clinicians to treat eventual other diseases according to a multidisciplinary approach [4,5,6].

Furthermore, dental caries, compromising oral health, can influence the general health of a subject. In fact, severe caries can change the quality of life of children and adolescents, causing pain, discomfort, acute and chronic infections, and loss of school days with consequent repercussions in learning and didactic training [7]. The Oral Health-Related Quality of Life (OHRQoL) is part of the general health of a subject and can be assessed by the way in which oral tissues and teeth influence physical health, psychological, and social well-being [8]. Several questionnaires that measure OHRQoL have been developed and validated in different languages and used for public health policies, research, and clinical practice [9]. The OHRQoL can be evaluated through standardized questionnaires such as the ECOHIS (Early Childhood Oral Health Impact Scale), which evaluates the OHRQoL in children under the age of six and the COHRQoL (Child Oral Health-Related Quality of Life), which measures the OHRQoL in children between the ages of six and 14. The COHRQoL includes two questionnaires: the PCPQ (Parental-Caregivers Perception Questionnaire) that evaluates the impact of oral health on the patient and the FIS (Family Impact Scale) that evaluates the impact of a patient’s oral health on the family [10,11].

In addition to the quality of life, dental caries and the related systemic inflammation can affect the nutrition and, consequently, the growth of a child [12]. However, the evidence on the probable effects of untreated dental caries on growth and health is poor.

The scientific evidence linking caries in primary teeth and growth is contradictory, both in terms of the presence and the direction of the association [13,14]. Some studies report a relationship between caries and reduced growth due to the influence that dental caries can have on the mechanisms that regulate and control growth [15]. Two theories can explain this relationship:

1. Inflammation and pain, associated with extensive untreated carious processes, directly affect a child’s ability to eat, causing malnutrition and consequently reduced growth [16]. Laboratory analysis (protein electrophoresis, C-reactive protein, REUMA test, ESR (erythrocyte sedimentation rate), etc.) may suggest the presence of an inflammatory process or the presence of a nonspecific response to an inflammatory situation that may involve the oral cavity [17].
2. Chronic dental infection, resulting from one or more untreated caries, may have indirect systemic effects. Three mechanisms have been suggested:
  - Interaction with the immune response. Cytokines (in particular IL-1) and other inflammatory factors that are released from damaged tissues during inflammatory processes, such as pulpits or chronic dental abscesses, suppress erythropoiesis and hemoglobin synthesis. This situation may determine anemia, bone remodeling influence, sleep, and food intake alterations [18,19]. In a 2013 study, Schroth et al. noted that children with severe caries had significantly lower values of ferritin (*p* = 0.003) than controls. The ability to recognize the warning signs of low iron levels may allow patients to receive treatments before more serious conditions can occur [20];
  - Interaction with the endocrine response. The interruption of slow-wave sleep, due to pain, can lead to insufficient secretion of aldosterol and growth hormone (GH) [21]. Furthermore, according to a study conducted by Roman et al. in 2009, the BMI modulates the response of IGF-I to GH, suggesting that the sensitivity of GH may be influenced by nutritional status in children [22]. According to some authors, there is a correlation between children who have untreated dental caries and BMI alteration [23];
  - Interaction with metabolism and homeostasis. Infection and related inflammation could lead to a low intake of micronutrients, determined by the increase in energy costs and metabolic demands, as well as by altered absorption [24]. Few studies have dealt with reduced food intake due to chewing difficulties in children suffering from destructive caries and the consequent lack of important vitamins and nutrients (Vitamin B12, Vitamin D, folate, triglycerides, cholesterol) [25]. A case-control study conducted by Schroth in 2013 showed that albumin levels are lower (*p* < 0.001) in children with numerous caries [20]. According to what was said by Collins et al. in 2001, albumin constitutes a long-term measure of the patient’s protein level and can, therefore, be used as an additional indicator of the overall nutritional status. An albumin deficiency associated with the deficiency of other substances in children with several complicated caries may indicate a serious nutritional deficiency [26].

To outline the nutritional and health conditions of children, the World Health Organization (WHO) recognizes growth as the best parameter. Anthropometry is the most universally applicable, inexpensive, and non-invasive method for assessing the size, proportions, and composition of the human body. The most frequent anthropometric values found in studies aimed at investigating a possible relationship between caries and growth are weight, height, and body mass index (BMI) [27,28,29]. To analyze the growth of a child, it is necessary not only to evaluate the individual values but to compare them to a population-based growth model; in this regard, percentile tables can be used. Several studies report that after dental care, the children presented, in addition to an improvement in the quality of life, an increase in the average values of growth parameters compared to the baseline [16,30].

In contrast to studies reporting a relationship between caries and poor growth, some studies have found no relationship between anthropometric values and carious disease [30,31,32,33] or reported that dental caries is related to being overweight [30,34,35].

The present study was conducted with the following objectives:

- to evaluate the possible improvement of the quality of life and the possible increase in weight, height, and BMI following dental treatment under general anesthesia in uncooperative pediatric patients;
- to assess the possible correlation between dental caries and poor quality of life;
- to assess the influence of the subject’s oral health status on the family’s quality of life;
- to evaluate the variation in the blood chemistry parameters involved in the growth process;
- to assess the consistency, the strength of association, the specificity, the temporality, and the coherence of the blood chemistry parameters and the possible variation of growth.

## 2. Materials and Methods

This pilot study was conducted, after having received approval from the Ethics Committee for Biomedical Activities of the University of Naples “Federico II”, Naples—Italy (Protocol No. 5/18), in accordance with the Declaration of Helsinki et al. [36].

For the study, 65 subjects (35 females and 30 males) were selected. The subjects intended to have conservative and/or endodontic and/or extractive dental treatment conducted under general anesthesia. These patients were uncooperative because of young age or for the presence of systemic pathologies that do not allow treatment in the clinic under conventional procedure or in mild and/or moderate sedation. All participants had equally poor oral health at baseline (number of teeth with severe caries with pulp involvement ≥ 3), and their medical conditions did not influence the study data. The selected patients were aged between three and 15 years old, and the study lasted for two years.

The patients were recruited by the Department of Pediatric Dentistry of the University “Federico II”, Naples—Italy, where dental visits were carried out by a dentist, according to the standard procedure. On this occasion, the purpose and modalities of the study were shown to parents and pediatric patients and they were asked to participate voluntarily in the study. The information and the consent to the processing of personal data and the informed consent to the medical act before proceeding to any type of intervention were given to adhere to the study.

Before dental treatment under general anesthesia, according to the standard care procedure, electrocardiogram, pediatric, cardiology, anesthesiology consultations, and blood chemistry tests were carried out. Concerning the blood chemistry test, in addition to the standard values, albumin, ferritin, ALP (alkaline phosphatase), cholesterol, triglycerides, PCR (C-reactive protein), ESR (erythrocyte sedimentation rate), vitamin D, vitamin B12, folate, and IGF-1 (insulin-like growth factor-1) were also studied as biochemical parameters to be evaluated for the purposes of the study.

In addition, the patients’ weight, height, and body mass index (BMI) were measured according to the standard care procedure. The weight was measured using an electronic scale, with minimum standard clothing and without shoes, and the height was measured using a stadiometer. With these data, the BMI was calculated. The three values thus obtained were reported in the percentile tables recommended by the CDC (Centers for the prevention and Control of Diseases).

To the parents, for the purposes of the study, the ECOHIS questionnaire (Early Childhood Oral Health Impact Scale), if the subject was aged between 4 and 6 years, or the COHRQoL questionnaire (Child Oral Health-Related Quality of Life), composed by the two sections PCPQ (Parental-Caregivers Perception Questionnaire) and FIS (Family Impact Scale), if the subject was older than 6 years of age, was delivered.

The ECOHIS questionnaire includes 14 questions, of which nine constitute the child section and five constitute the family section. The child section includes four areas (symptoms, activity, psychological well-being, and social well-being); while the family section includes two areas (distress and activities). The following scores were assigned to the possible answers: 0 = never; 1 = almost never; 2 = occasionally; 3 = often; 4 = very often; 5 = I do not know. The total score ranges from 0 to 56, where a higher result indicates a worse quality of life.

The PCPQ questionnaire includes 16 questions related to four areas (oral symptoms, functional limitations, emotional well-being, social well-being), to which possible answers the following score was assigned: 0 = never; 1 = almost never; 2 = occasionally; 3 = often; 4 = very often; 5 = I don’t know; two questions related to the global assessment, of which the first was assigned to the possible answers the following score: 0 = excellent; 1 = above the sufficiency; 2 = sufficient; 3 = insufficient; 4 = poor; and the second: 0 = anything; 1 = little; 2 = sufficiently; 3 = enough; 4 = a lot. The total score of the PCPQ questionnaire ranges from 0 to 72, where a higher result indicates a worse quality of life.

The FIS questionnaire includes 12 questions divided into four areas (parental/family activities, parental emotions, family conflicts, financial burdens), to which possible answers the following score was assigned: 0 = never; 1 = once or twice; 2 = sometimes; 3 = often; 4 = very often; 5 = I don’t know. The total score ranges from 0 to 48, where a higher result indicates a worse quality of life.

After one week, patients underwent conservative/endodontic and/or extractive treatments under general anesthesia according to the normal care procedure.

One week later, the patients were checked by a dentist to evaluate the healing outcomes and possible problems or symptoms according to the protocol. Eight months after the intervention, the parents, contacted by telephone, were invited to the Department of Pediatric Dentistry of the University “Federico II”, Naples—Italy, with their children for follow up. On the occasion of this meeting, a new registration of the weight, height, the calculation of the new BMI, and a new blood test were performed to evaluate the possible variation of the values recorded at the baseline.

Parents were again given one of the two questionnaires, according to the presented criteria, for the purposes of the study.

The results obtained were subjected to statistical processing using the Student’s t test in the SPSS v.17.0 (SPSS Inc. Chicago, IL, U.S.A) for Mac computerized program in order to assess the influence of dental caries on the quality of life.

For the questionnaires, the frequency of possible answers to the questions was calculated and the totals of the questionnaires were analyzed by comparing the value to the baseline and to the follow up. The total PCPQ and FIS scores were calculated by summing the scores for all 30 questions. The total scores of the PCPQ, the four areas of the PCPQ, the FIS, and the four areas of the FIS were then obtained.

To assess the impact of oral health on growth, the delta values of weight, height, and BMI percentiles were assessed, based on the variation of the values calculated at the baseline and at the follow up, assigning a score equal to 1 = improved, 2 = worsened, and 3 = unchanged in order to calculate the frequencies with the SPSS v.17.0 for Mac program. To assess the impact of oral health on the general state of health, the delta values of the blood chemistry values taken into consideration were calculated based on the variation of the values at the baseline and at the follow up, assigning a score equal to 1 = improved, 2 = worsened, and 3 = unchanged in order to calculate the frequencies with the SPSS v.17.0 for Mac program.

## 3. Results

Of the 65 candidates in the study, 22 subjects were not included because they expressed dissent to informed consent. Of the 43 subjects (average age at enrollment: 8.32 ± 3.17 years), 15 were younger than 6 years (average age: 5.2 ± 1.66 years) and 28 were older than 6 years (average age: 10 ± 2.82 years).

### 3.1. The Effect of Dental Treatment Under General Anesthesia on Quality of Life

The results of the ECHOIS questionnaire collected both at baseline and at the 8-month follow up are shown in Table 1.

The results of the PCPQ questionnaire collected both at baseline and at the 8-month follow up are shown in Table 2.

The results of the FIS questionnaire collected both at baseline and at the 8-month follow up are shown in Table 3.

Table 4 shows the total values of the ECOHIS test, the total values of the PCPQ test, of the FIS, of the PCPQ + FIS, and of the areas at the baseline and at the follow up, with the respective significance levels.

### 3.2. The Effect of Dental Treatment under General Anesthesia on Growth

Of the 43 subjects included in the study, eight patients did not participate to the follow up and only completed the questionnaires.

**Figure 1:**
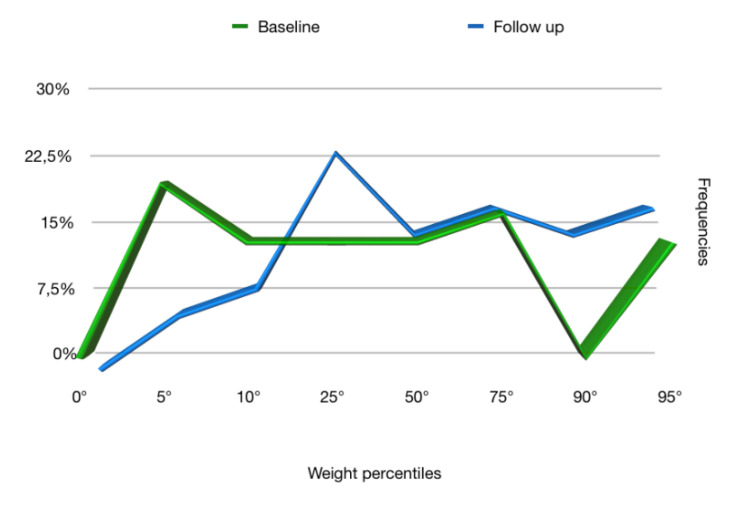
Weight percentiles at baseline and follow up.

**Figure 2:**
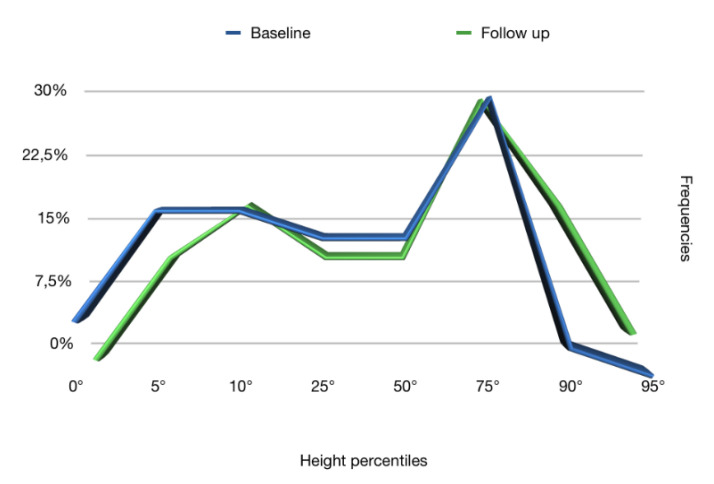
Height percentiles at baseline and follow up.

**Figure 3:**
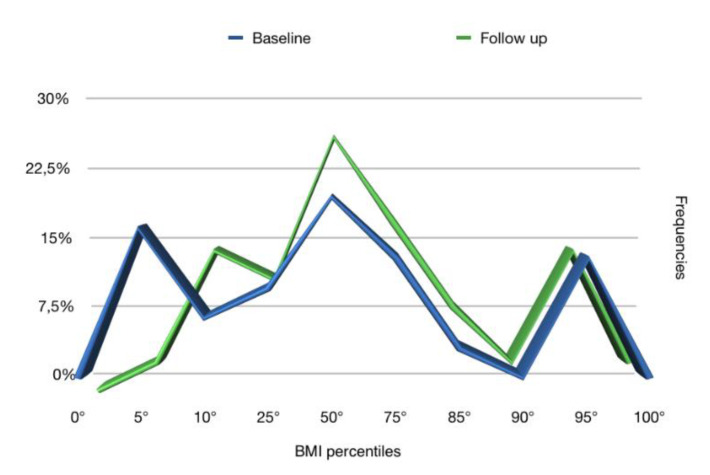
BMI percentiles at baseline and follow up.

The frequencies of the anthropometric values (weight, height, and BMI), obtained at the baseline and at the follow up, reported in terms of percentiles in Figure 1, Figure 2 and Figure 3, respectively, showed that the follow up curves shifted towards higher percentile values.

**Figure 4:**
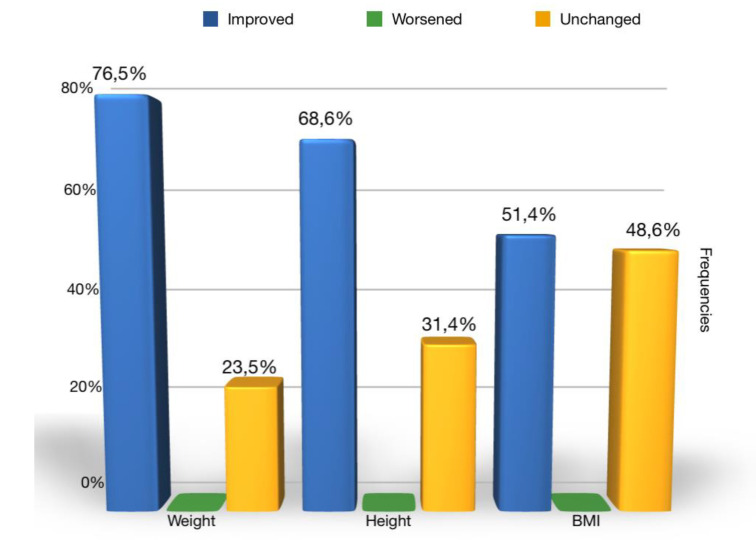
Percentile weight, height, and BMI evaluation at baseline and follow up.

Figure 4 reports the frequencies of the improvement, worsening, and invariability of anthropometric values (weight, height, and BMI).

### 3.3. The Effect of Dental Treatment under General Anesthesia on Blood Chemistry Parameters

**Figure 5:**
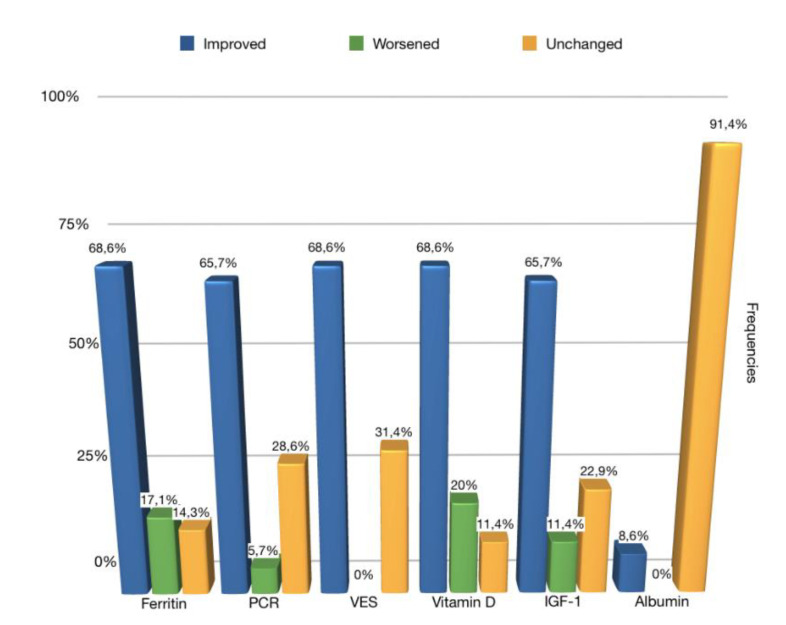
Relevant blood chemistry parameter evaluation at baseline and follow up.

As regards to the blood chemistry parameters examined, the relevant results obtained at the baseline and at the follow up are illustrated in Figure 5.

For the other observed blood chemistry parameters (ALP, triglycerides, total cholesterol, vitamin B12, and folate), a substantial variation was not observed in the entire sample.

## 4. Discussion

The analysis of the results shows a clear improvement in the quality of life, growth, and blood chemistry parameters with cessation of symptoms associated with the inflammatory and infectious state, a restoration of the correct masticatory function as well as a patient’s recovery under the social and psychological aspects. Families were also affected by a general improvement in the aspects analyzed by the questionnaires.

Regarding the impact of the dental treatment performed under general anesthesia on the quality of life of children suffering from severe caries using the OHRQoL questionnaires, the variation of the scores obtained at the baseline and at the follow up was evaluated.

Parents experienced great suffering due to the poor oral health of their children, who often complained of pain in the mouth and sensitivity during chewing; they had difficulty completing meals and problems with insomnia and behavior.

The results of the ECOHIS questionnaire showed clearly negative baseline values compared to those obtained at follow up. Regarding pain, at the baseline, 93% of the parents reported that their child had teeth, mouth or jaw pain frequently; at the follow up, 80% of the parents reported that their child “never” had pain. At the baseline, for 46.6% of the sample, it was reported that for their child, it was very difficult to eat, for 59.7% of the sample, the child showed evident difficulties in drinking. In a similar study by Low et al., the percentage was 43% [37]. At the follow up, in 100% of the sample, the parents reported that their child “never” had difficulty in eating and in 80% that they “never” had difficulty in drinking.

The difficulty in sleeping due to pain and frequent bleeding that emerged in other studies was also confirmed in this case [38]. In fact, at the baseline, 73.4% of patients had frequent problems during the night, but at the follow up, 0% of the sample reported difficulty sleeping due to dental pain. Severe caries, acute and chronic infections, and pain lead to the loss of school days which affects the learning and educational training of young patients. In the United States, dental care represents 117,000 h of school lost per 100,000 children [39]. In this study, it emerged that at the baseline, 86.6% of children had to leave school with a frequency that varied from “very often” to “occasionally”. At the follow up, 100% of the sample was no longer absent from school. In 40% of cases, parents noticed an improvement in the concentration and academic performance of their child. The impact on the quality of life of the family is equally important. Often having disturbed sleep because a child wakes up with dental pain symptoms becomes a burden on the family over time. Preparing different food or having a child who refuses to eat due to pain is equally disruptive to a family routine [40]. Due to the young age or other factors (e.g., disability), the subjects in question need to be accompanied by at least one parent to dental appointments. Many parents have difficulty organizing their time and are often absent from work. All this leads to a loss of earnings or the need to use one’s holidays. In this study, at the baseline, 86.7% of parents said they had been absent several times from their workplace and of these, 66.7% suffered a loss of earnings. This estimate is comparable to that of other studies [41,42,43]. An individual’s need for dental care often involves costs that affect the family economy. In fact, at the baseline, in 79.9% of the cases, the parents have already incurred expenses for the dental care of their children. After the dental treatment under general anesthesia, all the aspects analyzed by the questionnaire showed an improvement.

The results of the PCPQ and FIS questionnaire also showed clearly negative baseline values compared to those obtained at follow up. Regarding pain, at the baseline, 89.3% of the parents reported that their child had teeth, mouth or jaw pain frequently, while at the follow up, for 64.3% of the sample, the parents reported that their child “never” had more pain and for the remaining 35.7%, “almost never”. With regard to functional limitations, the results show that there was an improvement in masticatory and respiratory function. In fact, at the baseline, 82.1% of parents reported that their child frequently had difficulty chewing solid food, while at the follow up, 89.3% reported that their child “never” had more difficulty chewing food solid; in 78.6% of the sample, the parents reported that at the baseline, their child frequently breathed through the mouth, at the follow up, 71.4% of parents reported that their child no longer breathed through the mouth. Even the quality of sleep showed a clear improvement: at the baseline for 71.5% of parents, their child frequently had difficulty sleeping, while at the follow up, 92.9% of the patients “never” had difficulty sleeping. This factor also strongly influenced the improvement of the area of social and emotional well-being. With regard to the area of emotional well-being at the baseline, the average of the total answers was 10.60 ± 3.6 and at the follow up, 0.75 ± 1.17, highlighting how, after the dental treatment under general anesthesia, the children become less irritated or frustrated, nervous or scared, abandoning the sphere of negative emotions that affect their state of well-being. Regarding the area of social well-being, a systematic review concluded that it is the area least affected by the improvement [44]. The results of our study are in contrast with what was stated in the literature: in fact, the average of the total answers at the baseline was 8.96 ± 4.33, at follow up, the totality of the sample was in agreement with an equal score of 0. Regarding the area of global evaluation, meaningful are the answers to the question on the evaluation of the influence of the oral condition on the general well-being of the patients by the parents. In particular, both at the baseline and at the follow up, 92.9% of parents believed that oral health can influence the general well-being of their child. However, after the dental treatment under general anesthesia, the parents seemed to have a lower perception of the impact of oral health on general well-being; this could be explained by the achievement of the child’s state of health, abandoning the state of illness, for which health is no longer perceived as a need and assumes less relevance. Regarding the evaluation of oral health by parents, the data consistently reflected an assessment below the sufficiency at the baseline in 89.3% of cases and an evaluation of sufficient or above sufficiency in 100% of the cases at follow up.

The data that emerged from the FIS showed how the quality of life of parents and family clearly improved following the intervention, since, eliminating the cause of pain experienced by the patient, the child does not require more attention from the family, interrupting or disturbing the family activities or sleep quality. Regarding the area of parental emotions, as the state of illness disappeared, most parents did not feel unhappy or worried, and in particular, the sense of guilt for having neglected the oral health of the child changed from 82.1% of the sample of the parents at the baseline to 0% at the follow up. Also, the area of family conflicts showed a clear improvement between the baseline and the follow up. By improving the patient’s emotional well-being, the child was less inclined to create discussions or blame his parents, the general well-being of the family improved, and family discussions decreased in 100% of the sample. The well-being, after treatment, involves all the areas, including financial concerns, since there is no longer the need for care, and the parents do not have to bear expensive costs to improve their child’s oral health, causing financial difficulties for the family. The results obtained reflect the results reported by other studies in the literature [45,46,47].

Analysis of the Student’s t test indicated statistically significant differences for all the questionnaires and in all the areas of the questionnaires (*p* = 0.0001) between the baseline and the follow up. The results that emerge from this study indicate that dental rehabilitation under general anesthesia involves benefits not only for patients but for the entire family. Furthermore, the use of general anesthesia for non-cooperating pediatric patients, allowing dentists to perform all required treatments in a single session in hospital, provides fast oral health recovery.

Another aim of the study was to evaluate the level of association between caries and growth, since the data present in the literature are conflicting [12,13]. Sheiham et al. studied the effects of tooth decay on body weight, growth, and quality of life in preschool children, concluding that children with severe caries weighed less than the control group without caries. The same authors also reported weight gain and an improvement in the quality of life of patients after treatment [25,48].

In our study, 76.5% of patients at follow up achieved an increase in weight such as to shift to a higher percentile curve than the initial one. A significant increase was also recorded in height: 68.6% of patients achieved a height increase such as to fit into a higher percentile curve. As for the BMI, on the other hand, in 51.4% of cases, it increased and in 48.6% remained the same. In no case within the sample did the subject place himself at the follow up in a lower percentile curve than the baseline regarding weight, height, and BMI percentiles.

At the baseline, 22.9% of the sample had a weight less than or corresponding to the 5th percentile, a clear sign of insufficient growth, while at follow up, the percentage dropped to 5.7%. Similar data were also recorded for the height: at the baseline, 22.8% of the sample had a height lower or corresponding to the 5th percentile, while at follow up, the percentage dropped to 11.4%, exactly half. The BMI at the baseline was lower than the 5th percentile in 20% of patients, while at follow up, in 2.9%.

The results obtained in this pilot study suggest that dental rehabilitation, in association with an improvement in the quality of life of the patients, leads to pain relief, involves a restoration of masticatory function, and permits a more adequate and diversified diet, allowing, after eight months, an increase in weight, height, and BMI percentiles in more than half the sample.

Having verified the presence and the direction of the association between caries and reduced growth, the mechanisms that are involved in this phenomenon have been investigated, since in the literature there are no experimental studies that have examined, from the hematochemical point of view, the reasons for this increase in percentiles and post-dental recovery growth under general anesthesia.

The results of the present pilot study showed that in 65.7% of the cases there was an improvement in the PCR values recorded at the follow up compared to the baseline and in 68.6% of the cases, an improvement in the ESR values. These values, in accordance with an improvement in growth parameters, support the correlation between caries, inflammation, and reduced growth and show how inflammation and pain, associated with extensive untreated carious processes, affect a child’s ability to eat, causing malnutrition and consequently reduced growth [16].

The third blood chemistry parameter analyzed, ferritin, improved at the follow up compared to the baseline in 68.6% of the sample. These results are in line with the study conducted by Schrotch in 2013. The improvement in ferritin values can be explained by the resolution of inflammation since, as suggested by Means et al. in their studies conducted in 1992 and 2003, cytokines and other inflammation factors suppress erythropoiesis and hemoglobin synthesis and cause anemia. Iron deficiency can cause learning and memory deficits, decreased motor skills, and increased anxiety (all these aspects were evaluated by the OHRQoL questionnaire at the baseline and follow up). The results of the OHRQoL questionnaires showed, in fact, that there was an improvement in these parameters together with the ferritin values [18,19,20].

A fourth blood chemistry parameter analyzed in this study, which showed an appreciable improvement, was Vitamin D, which at follow up improved in 68.6% of the sample. At the baseline, vitamin D was deficient or insufficient (<20 ng/mL) in 48% of subjects, at the follow up, the percentage of subjects with values less than 20 ng/mL dropped to 20%. Low values at the baseline can be explained by the reduced food intake due to pain and functional limitations, according to the OHRQoL questionnaires scores, with a consequent reduction in the intake of nutrients and vitamins.

A fifth parameter among the blood chemistry values examined, which showed a significant improvement in the baseline values at follow up was IGF-1, which improved in 65.7% of subjects. Due to the change in sleep, reported by 86.7% of subjects under the age of six and by 71.5% of subjects over the age of six, caused by pain and infection, a reduction may occur in growth hormone secretion, as shown by the study conducted by Sheiham et al. in 2006 [21]. The alteration of BMI found in the study sample modulates the response of IGF-1 to growth hormone, supporting the hypothesis that growth hormone sensitivity can be influenced by the nutritional status of children [22]. The improvement in IGF-1 values is consistent with the increase in growth recorded at follow up and with the improvement in BMI percentile values by age.

The comparison of the albumin values between the baseline and follow up showed an improvement only in 8.6% of the sample, while in 91.4%, the values remained stable; in addition, only 2.9% of subjects had hypoalbuminemia at the baseline. These results are not in line with the studies of Schrotch et al. and of Clarke et al., who reported, respectively, that 18.6% and 15% of subjects with numerous complicated caries had hypoalbuminemia [20,49].

Finally, as for the limitations of this study, it was not possible to include a negative control group (non-cooperating patients with severe dental caries not undergoing treatment) for ethical reasons. In fact, the use of an untreated control group would have been appropriate methodologically, but not ethically. As a pilot study, the findings also identify a need to explore the interplay of diet and nutrient intake in the dental disease process. That is, poor nutrition could be a risk factor for dental decay and once decay becomes severe, a child may be even less likely to eat healthy foods due to dental pain.

## 5. Conclusions

It can be stated that dental treatment under general anesthesia significantly influences OHRQoL, growth, and blood chemistry parameters in uncooperative pediatric patients with compromised oral health. Based on the results obtained in this pilot study, other studies, involving a greater number of patients, will be needed to confirm the positive results obtained.

## Figures and Tables

**Table 1:** ECOHIS (Early Childhood Oral Health Impact Scale) questionnaire results.

ECOHIS	Never N 15		Almost Never N 15		Occasionally N 15		Often N15		Very Often N 15	
Questions	T0	T1	T0	T1	T0	T1	T0	T1	T0	T1
Children Section										
How often has your child had pain in his/her teeth, mouth, or jaws?	0	12 (80%)	0	3 (20%)	1 (6.7%)	0	6 (40%)	0	8 (53.3%)	0
How often has your child … due to dental problems or dental care?										
had difficulty drinking hot or cold drinks	0	12 (80%)	0	3 (20%)	6 (40%)	0	4 (26.7%)	0	5 (33.3%)	0
had difficulty eating some foods	0	15 (100%)	2 (13.3%)	0	6 (40%)	0	5 (33.3%)	0	2 (13.3%)	0
had difficulty pronouncing some words	2 (13.3%)	15 (100%)	6 (40%)	0	2 (13.3%)	0	3 (20%)	0	2 (13.3%)	0
missed preschool, day care or school	0	15 (100%)	2 (13.3%)	0	5 (33.3%)	0	6 (40%)	0	2 (13.3%)	0
had trouble sleeping	0	15 (100%)	2 (13.3%)	0	2 (13.3%)	0	7 (46.7%)	0	4 (26.7%)	0
been irritable or frustrated	0	12 (80%)	2 (13.3%)	3 (20%)	7 (46.7%)	0	3 (20%)	0	3 (20%)	0
avoided smiling or laughing	4 (26.7%)	15 (100%)	5 (33.3%)	0	6 (40%)	0	0	0	0	0
avoided talking	3 (20%)	15 (100%)	4 (26.7%)	0	7 (46.7%)	0	1 (6.7%)	0	0	0
Family Section										
How often have you or another family member … because of your child’s dental problems or dental care?										
been upset	1 (6.7%)	15 (100%)	3 (20%)	0	8 (53.3%)	0	3 (20%)	0	0	0
felt guilty	0	10 (66.7%)	6 (40%)	5 (33.3%)	5 (33.3%)	0	4 (26.7%)	0	0	0
taken time off from work	0	15 (100%)	2 (13.3%)	0	6 (40%)	0	4 (26.7%)	0	3 (20%)	0
suffered a loss of earnings while absent from work	3 (20%)	15 (100%)	2 (13.3%)	0	3 (20%)	0	6 (40%)	0	1 (6.7%)	0
How often has your child had dental problems or dental treatments that had a financial impact on your family?	0	15 (100%)	3 (20%)	0	8 (53.3%)	0	2 (13.3%)	0	2 (13.3%)	0

**Table 2:** PCPQ (Parental-Caregivers Perception Questionnaire) questionnaire results.

PCPQ	Never N 28		Almost Never N 28		Occasionally N 28		Often N 28		Very Often N 28	
Questions	T0	T1	T0	T1	T0	T1	T0	T1	T0	T1
During the past three months how often has your child had/was … due to tooth or mouth pain?										
Oral Symptoms										
bad breath	0	3 (10.7%)	3 (10.7%)	10 (35.7%)	9 (32.1%)	15 (53.6%)	9 (32.1%)	0	7 (25%)	0
pain in the teeth, lips, jaws, or mouth	1 (3.6%)	18 (64.3%)	2 (7.1%)	10 (35.7%)	10 (35.7%)	0	7 (25%)	0	8 (28.6%)	0
food stuck on the roof of the mouth	4 (14.3%)	28 (100%)	3 (10.7%)	0	6 (21.4%)	0	5 (17.9%)	0	10 (35.7%)	0
food caught in or between the teeth	2 (7.1%)	23 (82.1%)	3 (10.7%)	5 (17.9%)	6 (21.4%)	0	7 (25%)	0	10 (35.7%)	0
Functional Limitation										
difficulty biting or chewing firm foods	2 (7.1%)	25 (89.3%)	3 (10.7%)	1 (3.6%)	6 (21.4%)	0	10 (35.7%)	2 (7.1%)	7 (25%)	0
oral breathing	3 (10.7%)	20 (71.4%)	3 (10.7%)	5 (17.9%)	7 (25%)	3 (10.7%)	8 (28.6%)	0	7 (25%)	0
taken longer than others to eat a meal	3 (10.7%)	24 (85.7%)	3 (10.7%)	4 (14.3%)	7 (25%)	0	9 (32.1%)	0	6 (21.4%)	0
had trouble sleeping	4 (14.3%)	26 (92.9%)	4 (14.3%)	2 (7.1%)	5 (17.9%)	0	10 (35.7%)	0	5 (17.9%)	0
Emotional Wellbeing										
been irritable or frustrated	5 (17.9%)	20 (71.4%)	2 (7.1%)	8 (28.6%)	2 (7.1%)	0	7 (25%)	0	12 (42.9%)	0
been upset	5 (17.9)	23 (82.1)	6 (21.4)	5 (17.9)	3 (10.7)	0	4 (14.3)	0	10 (35.7)	0
been anxious or fearful	0	20 (71.4%)	4 (14.3%)	8 (28.6%)	4 (14.3%)	0	3 (10.7%)	0	17 (60.7%)	0
acted shy or embarrassed	2 (7.1%)	28 (100%)	4 (14.3%)	0	7 (25%)	0	9 (32.1%)	0	6 (21.4%)	0
Social Wellbeing										
missed school or pre-school	7 (25%)	28 (100%)	5 (17.9%)	0	5 (17.9%)	0	6 (21.4%)	0	5 (17.9%)	0
avoid smiling or laughing when around other children	3 (10.7%)	28 (100%)	6 (21.4%)	0	2 (7.1%)	0	8 (28.6%)	0	9 (32.1%)	0
had a hard time paying attention in school	4 (14.3%)	28 (100%)	4 (14.3%)	0	6 (21.4%)	0	8 (28.6%)	0	6 (21.4%)	0
not wanted to talk to other children	6 (21.4%)	28 (100%)	4 (14.3%)	0	4 (14.3%)	0	5 (17.9%)	0	8 (28.6%)	0
Global Evaluation										
Questions	Excellent		Above the Sufficiency		Sufficient		Insufficient		Poor	
How much would you rate your child’s teeth, lips, and mouth health?	0	1 (3.6%)	0	16 (57.1%)	3 (10.7%)	11 (39.3%)	13 (46.4%)	0	12 (42.9%)	0
Questions	Anything		Little		Sufficiently		Enough		A lot	
How much is your child’s general wellbeing affected by the condition of this teeth, lips, jaws or mouth?	0	0	2 (7.1%)	2 (7.1%)	7 (25%)	4 (14.3%)	7 (25%)	14 (50%)	12 (42.9%)	8 (28.6%)

**Table 3:** FIS (Family Impact Scale) questionnaire results.

FIS	Never N 28		Once or Twice N 28		Sometimes N 28		Often N 28		Very Often N 28	
Questions	T0	T1	T0	T1	T0	T1	T0	T1	T0	T1
During the past three months, because of your child’s teeth, lips, mouth, or jaws, how often …										
Parental/Family Activity										
Has your child required more attention from you or the other parent?	2 (7.1%)	22 (78.6%)	1 (3.6%)	6 (21.4%)	7 (25%)	0	9 (32.1%)	0	9 (32.1%)	0
Have you or the other parent had less time for yourselves or other family members?	2 (7.1%)	20 (71.4%)	1 (3.6%)	8 (28.6%)	9 (32.1%)	0	7 (25%)	0	9 (32.1%)	0
Has your sleep or that of the other parent been disturbed?	4 (14.3%)	19 (67.9%)	3 (10.7%)	9 (32.1%)	6 (21.4%)	0	9 (32.1%)	0	6 (21.4%)	0
Have family activities been interrupted?	9 (32.1%)	24 (85.7%)	6 (21.4%)	4 (14.3%)	5 (17.9%)	0	4 (14.3%)	0	4 (14.3%)	0
Parental Emotion										
Have you or the other parent been upset?	1 (3.6%)	27 (96.4%)	3 (10.7%)	1 (3.6%)	8 (28.6%)	0	10 (35.7%)	0	6 (21.4%)	0
Have you or the other parent worried that your child will have fewer life opportunities?	5 (17.9%)	28 (100%)	4 (14.3%)	0	3 (10.7%)	0	9 (32.1%)	0	7 (25%)	0
Have you or the other parent felt guilty?	5 (17.9%)	28 (100%)	6 (21.4%)	0	6 (21.4%)	0	8 (28.6%)	0	3 (10.7%)	0
Family Conflict										
Has your child argued with you or the other parent?	5 (17.9%)	28 (100%)	4 (14.3%)	0	7 (25%)	0	6 (21.4%)	0	6 (21.4%)	0
Has your child been jealous of you or other family members?	6 (21.4%)	28 (100%)	1 (3.6%)	0	6 (21.4%)	0	7 (25%)	0	8 (28.6%)	0
Has your child’s condition caused disagreement or conflict in the family?	5 (17.9%)	28 (100%)	4 (14.3%)	0	6 (21.4%)	0	5 (17.9%)	0	8 (28.6%)	0
Has your child blamed you or the other parent?	7 (25%)	26 (92.9%)	4 (14.3%)	2 (7.1%)	6 (21.4%)	0	6 (21.4%)	0	5 (17.9%)	0
Financial Burden										
Has your child’s condition caused financial difficulties for your family?	10 (35.7%)	26 (92.9%)	2 (7.1%)	1 (3.6%)	6 (21.4%)	1 (3.6%)	3 (10.7%)	0	7 (25%)	0

**Table 4:** Student’s t test for ECOHIS, PCPQ, and FIS analysis.

ECOHIS, PCPQ, and FIS	N Sample	T0 Average (DS)	T1 Average (DS)	*p* Value
ECOHIS	15	31.5 (4.2)	0.93 (1.28)	0.0001
PCPQ	28	46.3 (11.9)	7.93 (2.83)	0.0001
Oral symptoms	28	10.61 (3.46)	1.96 (1.04)	0.0001
Functional limitation	28	9.78 (2.84)	0.86 (1.29)	0.0001
Emotional wellbeing	28	10.60 (3.6)	0.75 (1.17)	0.0001
Social wellbeing	28	8.96 (4.33)	0 (0)	0.0001
Global evaluation	28	6.36 (1.39)	4.35 (1.13)	0.0001
FIS	28	26.78 (11.9)	1.36 (1.42)	0.0001
Parental/family activity	28	9.43 (3.99)	0.96 (1.24)	0.0001
Parental emotion	28	6.85 (3.00)	0.03 (0.19)	0.0001
Family conflict	28	8.50 (4.79)	0.14 (0.52)	0.0001
Financial burden	28	1.83 (1.63)	0.22 (0.83)	0.0001
PCPQ+FIS	28	73.11 (22.73)	9.28 (3.73)	0.0001
